# Syndromic Surveillance of Suicidal Ideation and Self-Directed Violence — United States, January 2017–December 2018

**DOI:** 10.15585/mmwr.mm6904a3

**Published:** 2020-01-31

**Authors:** Marissa L. Zwald, Kristin M. Holland, Francis B. Annor, Aaron Kite-Powell, Steven A. Sumner, Daniel A. Bowen, Alana M. Vivolo-Kantor, Deborah M. Stone, Alex E. Crosby

**Affiliations:** ^1^Division of Violence Prevention, National Center for Injury Prevention and Control, CDC; ^2^Division of Health Informatics and Surveillance, Center for Surveillance, Epidemiology, and Laboratory Services, CDC; ^3^Division of Overdose Prevention, National Center for Injury Prevention and Control, CDC.

Suicide is a growing public health problem in the United States, claiming approximately 47,000 lives in 2017 (*1*). However, deaths from suicide represent only a small part of a larger problem because each year millions of persons experience suicidal ideation and engage in suicidal and nonsuicidal self-directed violence, both risk factors for suicide (*2*). Emergency departments (EDs) are an important setting for monitoring these events in near real time (*3*–*5*). From 2001 to 2016, ED visit rates for nonfatal self-harm increased 42% among persons aged ≥10 years (*1*). Using data from CDC’s National Syndromic Surveillance Program (NSSP), ED visits for suicidal ideation, self-directed violence, or both among persons aged ≥10 years during January 2017–December 2018 were examined by sex, age group, and U.S. region. During the 24-month period, the rate of ED visits for suicidal ideation, self-directed violence, or both increased 25.5% overall, with an average increase of 1.2% per month. Suicide prevention requires comprehensive and multisectoral approaches to addressing risk at personal, relationship, community, and societal levels. ED syndromic surveillance data can provide timely trend information and can support more targeted and prompt public health investigation and response. CDC’s Preventing Suicide: A Technical Package of Policy, Programs, and Practices includes tailored suicide prevention strategies for health care settings (*6*).

CDC’s NSSP BioSense Platform,* a national public health surveillance system, was used to identify ED visits for this study. At the time of this investigation,^†^ NSSP included data from approximately 65% of visits at facilities categorized as EDs (i.e., urgent care and outpatient facilities were excluded) from 55 jurisdictions in 45 states.^§^ The Electronic Surveillance System for the Early Notification of Community-based Epidemics (ESSENCE) tool in the BioSense Platform was used to analyze ED visits. In collaboration with CDC, the NSSP Community of Practice Syndrome Definition Committee developed a definition to identify ED visits involving suicidal ideation, self-directed violence, or both, which combines clinical presentation and Boolean operators (e.g., hanging, laceration, or overdose attempt) and diagnosis codes associated with suicidal ideation, self-directed violence, or both.^¶^ The definition is designed to query patients’ chief complaint history, discharge diagnosis, and admission reason code and description fields and includes common misspellings of suicide-related terms, while excluding visits in which a patient “denies suicidal ideation” or “is not suicidal.” The syndrome definition used for this investigation does not differentiate between suicidal ideation and self-directed violence, nor the method of self-directed violence (*7*). The composite measure used in this investigation was the first syndrome definition ever developed by the NSSP Community of Practice Syndrome Definition Committee and CDC to capture ED visits broadly related to suicidal ideation, self-directed violence, or both. More specific syndrome definitions that separately assess ED visits related to suicidal ideation, self-directed violence, or specific mechanisms of self-directed violence are in development.

Monthly ED visits involving suicidal ideation, self-directed violence, or both per 100,000 ED visits among persons aged ≥10 years during January 2017–December 2018 were computed overall and stratified by sex, age group, and U.S. region.** Rates were calculated by dividing the number of ED visits related to suicidal ideation, self-directed violence, or both by the total number of ED visits recorded in ESSENCE each month, multiplied by 100,000. Percentage changes in the monthly rate for ED visits for suicidal ideation, self-directed violence, or both overall and for each stratum were examined. Estimates of average monthly percentage change were calculated using Joinpoint regression with Joinpoint software (version 4.7.0.0; National Cancer Institute).^††^ P-values <0.05 were considered statistically significant.

During January 2017–December 2018, among approximately 163 million ED visits assessed in NSSP, a total of 2,123,614 involved suicidal ideation, self-directed violence, or both (1,300.6 per 100,000 ED visits). During the same period, the rate of ED visits involving suicidal ideation, self-directed violence, or both increased 25.5%, with an average increase of 1.2% per month (Table). Both sexes experienced significant increases during this period: the rate increased 22.7% for females and 27.6% for males (Table) (Figure 1). Among females, ED visit rates involving suicidal ideation, self-directed violence, or both significantly increased among those aged 10–19 years (33.7% increase), 40–59 years (17.6%), and ≥60 years (29.0%). Females aged 20–39 years did not experience a significant increase in ED visit rate for suicidal ideation, self-directed violence, or both. Among males, all age groups experienced significant increases in ED visit rates related to suicidal ideation, self-directed violence, or both during January 2017–December 2018, including those aged 10–19 years (62.3%), 20–39 years (29.1%), 40–59 years (20.4%), and ≥60 years (36.7%). For both females and males aged 10–19 years, a seasonal pattern in ED visits for suicidal ideation, self-directed violence, or both was observed, with the lowest proportion of visits occurring during summer months. Three of five U.S. regions experienced significant increases in these ED visit rates: the Midwest (33.8%), Northeast (16.0%), and West (13.3%) (Table) (Figure 2). Among females, rates of ED visits related to suicidal ideation, self-directed violence, or both significantly increased in the Midwest (28.7%), West (14.7%), and Northeast (13.6%). Among males, rates of ED visits related to suicidal ideation, self-directed violence, or both significantly increased in all U.S. regions except the Southwest (Midwest, 38.7%; Southeast, 33.5%; Northeast, 17.7%; and West, 11.1%). Rates were consistently highest in the West for both females and males.

**TABLE:** Changes in monthly rate* of ED visits related to suicidal ideation, self-directed violence, or both, by sex, age group, and U.S. region^†^ — National Syndromic Surveillance Program, United States, January 2017–December 2018^§^

Characteristic	% Change			Average monthly % change (95% CI)
	From Jan 2017 to Dec 2017	From Jan 2018 to Dec 2018	From Jan 2017 to Dec 2018	
**Age group (yrs)**				
**Both sexes**				
**Overall**	**8.2^¶^**	**13.7^¶^**	**25.5^¶^**	**1.2 (1.0 to 1.5)^¶^**
10–19	17.2	17.9	43.4^¶^	1.7 (1.0 to 2.4)^¶^
20–39	10.7^¶^	13.7^¶^	28.5^¶^	1.2 (0.4 to 2.0)^¶^
40–59	5.6	15.2^¶^	19.7^¶^	0.9 (0.1 to 1.7)^¶^
≥60	11.0^¶^	23.3^¶^	33.4^¶^	1.3 (0.4 to 2.2)^¶^
**Females**				
**Overall**	**5.6^¶^**	**11.2**	**22.7^¶^**	**1.2 (0.9 to 1.5)^¶^**
10–19	11.6	14.3	33.7^¶^	1.4 (0.7 to 2.1)^¶^
20–39	8.4	12.5^¶^	27.1	1.1 (−0.1 to 2.4)
40–59	3.7	12.8^¶^	17.6^¶^	0.9 (0.2 to 1.5)^¶^
≥60	7.1	23.6^¶^	29.0^¶^	1.2 (0.1 to 2.4)^¶^
**Males**				
**Overall**	**10.3^¶^**	**15.7^¶^**	**27.6^¶^**	**1.2 (1.1 to 1.4)^¶^**
10–19	28.2^¶^	24.4	62.3^¶^	2.2 (1.5 to 2.9)^¶^
20–39	12.3^¶^	13.8^¶^	29.1^¶^	1.4 (0.7 to 2.0)^¶^
40–59	6.5^¶^	16.1^¶^	20.4^¶^	0.9 (0.4 to 1.5)^¶^
≥60	14.1^¶^	23.0^¶^	36.7^¶^	1.4 (0.6 to 2.2)^¶^
**U.S. region^†^**				
**Both sexes**				
Northeast	10.0^¶^	3.8	16.0^¶^	1.1 (0.8 to 1.3)^¶^
Southeast	8.2	25.8^¶^	30.2	1.5 (0.0 to 3.0)
Southwest	−7.9	13.8^¶^	9.6	0.6 (−0.8 to 2.0)
Midwest	10.0^¶^	15.7^¶^	33.8^¶^	1.3 (1.1 to 1.6)^¶^
West	0.1	7.3^¶^	13.3^¶^	0.5 (0.3 to 0.8)^¶^
**Females**				
Northeast	9.2	−0.1	13.6^¶^	1.0 (0.6 to 1.4)^¶^
Southeast	4.3	24.5^¶^	26.1	1.2 (−0.5 to 3.1)
Southwest	−12.0	10.1	5.0	0.4 (−1.3 to 2.1)
Midwest	4.3^¶^	14.5^¶^	28.7^¶^	1.3 (1.0 to 1.7)^¶^
West	1.0	5.2^¶^	14.7^¶^	0.5 (0.2 to 0.8)^¶^
**Males**				
Northeast	10.2^¶^	7.0^¶^	17.7^¶^	1.1 (0.9 to 1.3)^¶^
Southeast	11.4	26.7^¶^	33.5^¶^	1.6 (0.1 to 3.0)^¶^
Southwest	−3.7	16.6^¶^	13.6	0.6 (−0.5 to 1.7)
Midwest	15.5^¶^	16.6^¶^	38.7^¶^	1.3 (1.1 to 1.6)^¶^
West	−1.1	8.9^¶^	11.1^¶^	0.5 (0.2 to 0.9)^¶^

**Abbreviations:** CI = confidence interval; ED = emergency department.

* Per 100,000 visits. Calculated as number of ED visits related to suicidal ideation, self-directed violence, or both divided by the total number of ED visits for each month and multiplied by 100,000. Percentage change in rates were determined by subtracting the number during the previous month from the number during the current month, then dividing by the previous month’s number multiplied by 100%.

^†^ The *Northeast* region includes U.S. Department of Health and Human Services (HHS) Region 1 (Maine, Massachusetts, New Hampshire, Rhode Island, and Vermont), HHS Region 2 (New Jersey and New York), and HHS Region 3 (District of Columbia, Maryland, Pennsylvania, Virginia, and West Virginia); the *Southeast* region includes HHS Region 4 (Alabama, Florida, Georgia, Kentucky, Mississippi, North Carolina, South Carolina, and Tennessee); the *Southwest* region includes HHS Region 6 (Arkansas, Louisiana, New Mexico, and Texas); the Midwest region includes HHS Region 5 (Indiana, Illinois, Michigan, Minnesota, Ohio, and Wisconsin) and HHS Region 7 (Iowa, Kansas, Missouri, and Nebraska); and the *West* region includes HHS Region 8 (Colorado, Montana, North Dakota, and Utah), HHS Region 9 (Arizona, California, and Nevada), and HHS Region 10 (Alaska, Idaho, Oregon, and Washington).

^§^ Data are current as of February 8, 2019.

^¶^ Statistically significant (p<0.05).

**FIGURE 1:**
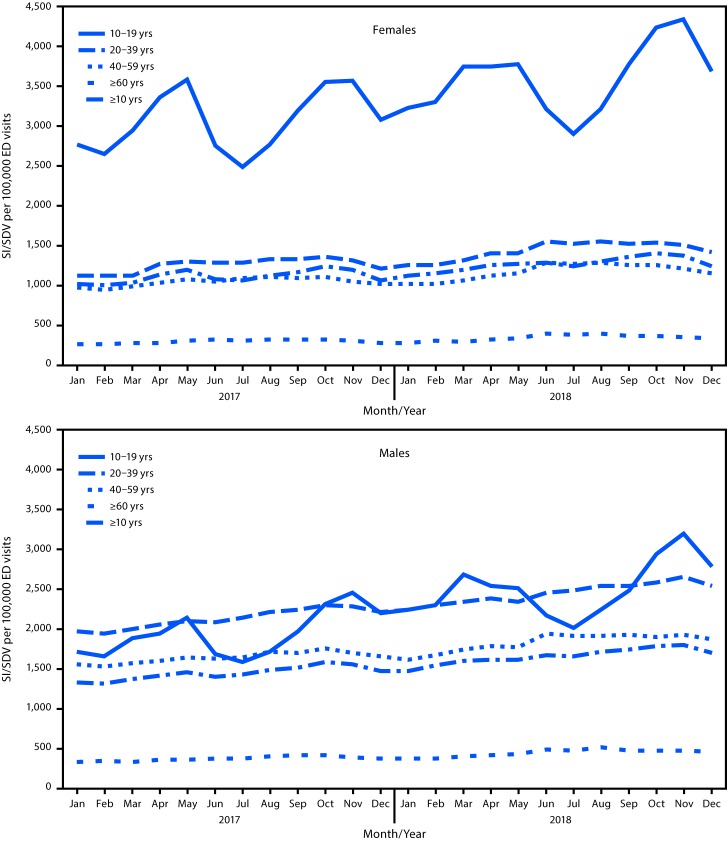
Monthly rate* of emergency department (ED) visits related to suicidal ideation (SI), self-directed violence (SDV), or both, by sex and age group — National Syndromic Surveillance Program, United States, January 2017–December 2018^†^ * Per 100,000 visits. Calculated as number of ED visits related to SI, SDV, or both, divided by the total number of ED visits for each month and multiplied by 100,000. ^†^ Data are current as of February 8, 2019.

**FIGURE 2:**
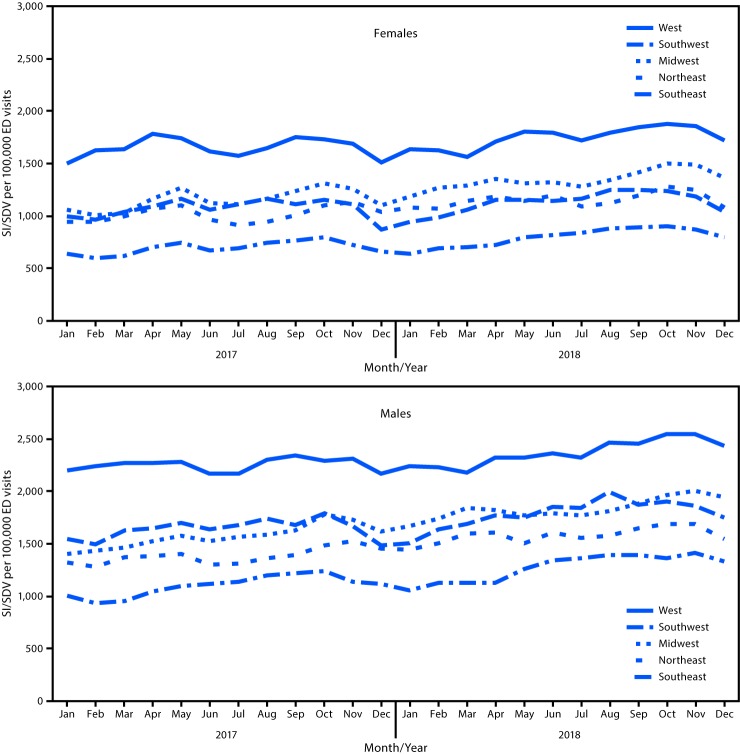
Monthly rate* of emergency department (ED) visits related to suicidal ideation (SI), self-directed violence (SDV), or both, by sex and region^†^ — National Syndromic Surveillance Program, United States, January 2017–December 2018^§^ * Per 100,000 visits. Calculated as number of ED visits related to SI, SDV, or both, divided by the total number of ED visits for each month and multiplied by 100,000. ^†^ *Northeast*: U.S. Department of Health and Human Services (HHS) Region 1 (Maine, Massachusetts, New Hampshire, Rhode Island, and Vermont), HHS Region 2 (New Jersey and New York), and HHS Region 3 (District of Columbia, Maryland, Pennsylvania, Virginia, and West Virginia); *Southeast*: HHS Region 4 (Alabama, Florida, Georgia, Kentucky, Mississippi, North Carolina, South Carolina, and Tennessee); *Southwest*: HHS Region 6 (Arkansas, Louisiana, New Mexico, and Texas); *Midwest*: HHS Region 5 (Indiana, Illinois, Michigan, Minnesota, Ohio, and Wisconsin) and HHS Region 7 (Iowa, Kansas, Missouri, and Nebraska); *West*: HHS Region 8 (Colorado, Montana, North Dakota, and Utah), HHS Region 9 (Arizona, California, and Nevada), and HHS Region 10 (Alaska, Idaho, Oregon, and Washington). ^§^ Data are current as of February 8, 2019.

## Discussion

Syndromic surveillance data from NSSP indicate a significant 25.5% increase in the rate of ED visits involving suicidal ideation, self-directed violence, or both during January 2017–December 2018, with substantial increases occurring in younger age groups. Other studies have described increases in rates among younger age groups in earlier years (*1*,*8*), and the current trends suggest persistence of these increases into 2018. The large increase in ED visits related to suicidal ideation, self-directed violence, or both for females aged 10–19 years suggests that a previously documented increase might also be continuing. For example, research has shown that from 2009 to 2015, ED visits for self-inflicted injury increased 18.8% among females aged 10–14 years and 7.2% per year among females aged 15–19 years (*8*). Among the demographic groups examined, males aged 10–19 years experienced the largest significant increase during this period (62.3%), which diverges from earlier studies showing a stable trend for younger males from 2001 to 2015, according to data from the National Electronic Injury Surveillance System–All Injury Program (NEISS-AIP) (*1*,*8*). One potential reason for these differences might be that NEISS-AIP only measures a person’s index visit, whereas NSSP records all ED visits from participating sites. Continued monitoring of trends in ED visits related to suicidal ideation, self-directed violence, or both using data from NSSP and other surveillance systems could help elucidate these differences by sex over time.

With respect to geographic variation, the largest increases in rates of ED visits related to suicidal ideation, self-directed violence, or both were observed among males in the Midwest (38.7%), males in the Southeast (33.5%), and females in the Midwest (28.7%), compared with other regions. However, rates were consistently highest in the West, as has been previously reported (*9*,*10*). The seasonal variation of youth ED visits involving suicidal ideation, self-directed violence, or both observed in the present study demonstrates the need for additional research examining the relationship between school-related factors and suicidal ideation, self-directed violence, or both and highlights opportunities for improved hospital capacity management during months with higher prevalence. Future research should assess geographic and temporal variations in suicide-related ED visits and the risk for dying by suicide after ED screening and presentation of suicidal thoughts or behaviors. Research examining variation in the impact of policies, socioeconomic risk factors, and access to lethal means across the United States on nonfatal suicide-related outcomes is needed (*6*).

The findings in this report are subject to at least five limitations. First, facility participation in NSSP can vary across months. To account for these changes, monthly trends in ED visits for suicidal ideation, self-directed violence, or both were assessed as a percentage of the total number of ED visits for each month. The monthly rates of ED visits for suicidal ideation, self-directed violence, or both calculated for this study served as a proxy indicator for changes in suicide risk but could be influenced by changes in the denominator or characteristics of the populations served by participating facilities. Second, results are not generalizable to facilities not participating in NSSP. Data from NSSP facilities are also not nationally representative, nor representative of each U.S. region, and variations in ED visits related to suicidal ideation, self-directed violence, or both occurring nationally and by U.S. region could reflect differences in the participating facilities contributing data to the system. Third, the syndrome definition used in this study might under- or overestimate ED visits related to suicidal ideation, self-directed violence, or both because of differences in coding, reporting, and availability of chief complaint text or discharge diagnosis data between jurisdictions or over time. The syndrome definition does not distinguish between incident and recurrent ED visits for suicidal ideation and self-directed violence, and it does not differentiate between suicidal ideation and self-directed violence. Therefore, the proportion of ED visits related to suicidal ideation, self-directed-violence, or the specific method of self-directed violence that contributed to the overall number of suicide-related ED visits in this investigation is unknown. Fourth, syndromic surveillance data used were transmitted to NSSP in near real time and are not considered finalized data sets. Thus, the reported findings should not be interpreted as exact case counts of suicidal ideation, self-directed violence, or both. Finally, without state or local context on the events, patterns, or behaviors of health systems and their patients, aggregating state and local syndromic surveillance data to the national or regional level might have less utility than would a methodology incorporating this local-level information into an early warning system for unusual patterns or potential clusters of nonfatal suicide-related outcomes (*3*–*5*).

Despite these limitations, these data identify important trends and variations across demographic and geographic groups and highlight the potential value of syndromic surveillance data to assist states and communities in detecting suicide-related events at more detailed geographic levels, thus facilitating more rapid and targeted public health prevention and response efforts. States and communities can also use resources such as CDC’s Preventing Suicide: A Technical Package of Policy, Programs, and Practices to guide suicide prevention initiatives. The CDC technical package includes seven strategies designed to help states and communities implement comprehensive suicide prevention efforts: strengthening economic supports, strengthening access and delivery of suicide care, creating protective environments, promoting connectedness, teaching coping and problem-solving skills, identifying and supporting persons at risk, and lessening harms and preventing future risk (*6*).

**Summary**

**What is already known about this topic?**

From 2001 to 2016, emergency department (ED) visit rates for nonfatal self-harm, which is associated with increased suicide risk, increased 42% among persons aged ≥10 years.

**What is added by this report?**

Analysis of CDC's National Syndromic Surveillance Program data showed that ED visit rates related to suicidal ideation, self-directed violence, or both increased 25.5% overall, with an average increase of 1.2% per month, nationwide during January 2017–December 2018.

**What are the implications for public health practice?**

ED syndromic surveillance data can provide timely suicidal ideation and self-directed violence trend information and can support more targeted and prompt public health investigation and response. CDC’s Preventing Suicide: A Technical Package of Policy, Programs, and Practices includes tailored suicide prevention strategies for health care settings.

## Footnotes

* NSSP’s BioSense platform was established in 2003 as a national public health surveillance system for early detection and rapid assessment of bioterrorism-related events and has expanded to track infectious diseases and injuries. https://www.cdc.gov/nssp/biosense/index.html.

† Data are current as of February 8, 2019.

§ Availability and completeness of chief complaint text and discharge diagnosis codes of ED visits reported in NSSP, which can also vary across months and by U.S. Department of Health and Human Services (HHS) region, can affect the ability of the syndrome definition to detect ED visits related to suicidal ideation, self-directed violence, or both. During the study period, completeness of chief complaint text was 87.6%, and completeness of discharge diagnosis code data was 62.3%.

¶ *International* *Classification of Diseases*, *Tenth Revision*, *Clinical Modification;* *International Classification of Diseases*, *Ninth Revision*, *Clinical Modification*; and Systematized Nomenclature of Medicine Clinical Terms discharge diagnosis codes associated with suicidal ideation, self-directed violence, or both were included in the syndrome definition..

** States listed are within the HHS regions that shared data with NSSP and had data available for the study period at the time of data analysis. In addition, some of the states listed do not provide data for the entire state. The *Northeast* region includes HHS Region 1 (Maine, Massachusetts, New Hampshire, Rhode Island, and Vermont), HHS Region 2 (New Jersey and New York), and HHS Region 3 (District of Columbia, Maryland, Pennsylvania, Virginia, and West Virginia); the *Southeast* region includes HHS Region 4 (Alabama, Florida, Georgia, Kentucky, Mississippi, North Carolina, South Carolina, and Tennessee); the *Southwest* region includes HHS Region 6 (Arkansas, Louisiana, New Mexico, and Texas); the *Midwest* region includes HHS Region 5 (Indiana, Illinois, Michigan, Minnesota, Ohio, and Wisconsin) and HHS Region 7 (Iowa, Kansas, Missouri, and Nebraska); and the *West* region includes HHS Region 8 (Colorado, Montana, North Dakota, and Utah), HHS Region 9 (Arizona, California, and Nevada), and HHS Region 10 (Alaska, Idaho, Oregon, and Washington).

†† https://surveillance.cancer.gov/joinpoint.
